# Latent class instrumental variables and the monotonicity assumption

**DOI:** 10.1186/s12982-020-00088-8

**Published:** 2020-03-19

**Authors:** Stuart G. Baker

**Affiliations:** Biometry Research Group, National Cancer Institute, Bethesda, MD 20892 USA

**Keywords:** Instrumental variables, Complier average causal effect, CACE, Local average treatment effect (LATE)

## Abstract

A key aspect of the article by Lousdal on instrumental variables was a discussion of the monotonicity assumption. However, there was no mention of the history of the development of this assumption. The purpose of this letter is to note that Baker and Lindeman and Imbens and Angrist independently introduced the monotonicity assumption into the analysis of instrumental variables. The letter also places the monotonicity assumption in the context of the method of latent class instrumental variables.

Lousdal [1] reviewed four basic assumptions of instrumental variable methodology and noted that the fourth basic assumption of monotonicity has received little attention in the literature. I would like to call attention to an omission in the Lousdal review, namely that the original work on the monotonicity assumption was not cited. Baker and Lindeman [2] and Imbens and Angrist [3] independently formulated an instrumental variables approach with the monotonicity assumption, which Imbens and Angrist called “monotonicity”. Baker et al. [4] provided historical background to the development of this methodology. They also coined the term latent class instrumental variables for an instrumental variable methodology involving randomization group or time period (treated like a randomization group) as the instrumental variable and having the following three characteristics. First, there are four latent classes (often called always-taker, never-takers compliers, and defiers [5]) based on treatment received if assigned to each group or time period. Second, the exclusion restriction assumption says that randomization group or time period does not affect the probability of outcome among always-takers and never-takers. Third, the monotonicity assumption specifies no defiers. As noted by Lousdal [1], with an instrumental variable of randomization group, the assumptions of relevance and exchangeability hold. Under these assumptions, the method of latent class instrumental variables estimates the effect of treatment among the latent class of compliers, which has various names including the effect of treatment received, [2] the local average treatment effect (LATE), [3] and the complier average causal effect (CACE) [6]. The terms LATE and CACE generally refer to differences in outcome among compliers. The method of latent class instrumental variables also estimates the relative risk for outcomes among compliers [7].

## Data Availability

Not applicable.
